# YCl_3_ Promotes Neuronal Cell Death by Inducing Apoptotic Pathways in Rats

**DOI:** 10.1155/2017/2183658

**Published:** 2017-02-23

**Authors:** Yechun Ding, Yuantong Tian, Zhaoyi Zeng, Ping Shuai, Haiying Lan, Xianshen Zhu, Yi Zhong, Longhuo Wu, Xiaona Fan

**Affiliations:** ^1^College of Pharmacy, Gannan Medical University, Ganzhou 341000, China; ^2^School of Basic Medicine, Gannan Medical University, Ganzhou 341000, China; ^3^The Third Affiliated Hospital, Gannan Medical University, Ganzhou 341000, China; ^4^Research Department, Gannan Medical University, Ganzhou 341000, China

## Abstract

The pollutants rare earth elements (REEs) have posed great threats to human health. To investigate the cytotoxicity of yttrium (Y), a model that rats have free access to water containing YCl_3_ for 6 months is utilized. The results showed that YCl_3_ treatment promoted neuronal cell apoptosis by upregulating the proapoptotic factors Bax, caspase-3, Cyto* c*, and DAPK and by downregulating the antiapoptotic factors Bcl-2 and XIAP at both mRNA and protein levels. Conclusively, YCl_3_ exhibited cytotoxicity and promoted neuronal cell death by the induction of apoptotic pathways.

## 1. Introduction

Metal ions from drinking water and food have been extensively reported to be of importance in affecting our health. Rare earth elements (REEs) are widely used in medical, agricultural, and industrial fields [1]. Many REEs show medicinal benefits for human health [2]. ^90^Yttrium (^90^Y), a high energy beta particle-radiating radioisotope, has been comprehensively used in clinical situation since 1969. ^90^Y therapy has two available radioembolization devices, including glass microspheres called TheraSphere and resin microspheres called SIR-Sphere [3], for management of colorectal cancer and liver metastases [4]. Yttrium oxide nanoparticle is known as free radicals scavenger to protect undifferentiated PC12 cells from oxidative stress-induced apoptosis [5].

Recently, REEs are considered as emerging trace pollutants, indicating that REEs are relatively safe [6]. Although increasing evidence shows that REEs promote the production in agriculture and poultry industry, the negative impacts of REEs accumulation in human body through the food chain have been confirmed [7]. Animal studies have also demonstrated that REEs may produce severe adverse effects by inducing imbalance of oxidative stress in human body, and leading to development of hypertension [8]. Long-term administration of REEs may cause negative outcomes. 15 REEs, including yttrium, have been biologically evaluated and proved for risk in inducing hypertension in housewives [9]. The possible explanation might be associated with the disturbance of REEs in redox balance, causing chronic oxidative stress damage and cell apoptosis [10]. Another theory may be related to their competition with those important nutritional ions, such as Ca^2+^, Zn^2+^, Mg^2+^, and Cu^2+^ [11]. The exact mechanism is still unclear.

Apoptosis occurs extensively during nervous system development and is important for the establishment of neuronal populations with correct sizes. In addition, apoptosis is one of the major mechanisms of neuronal death in pathological development. Brain functional alternation or damage may be induced by pathological neuronal apoptosis. Methamphetamine (METH) induced neuronal apoptosis may impair adult hippocampal neurogenesis, which is related to maintenance of hippocampal-dependent learning and memory [12]. Similarly, neuronal apoptosis in hippocampus, cortex, and striatum is involved in high altitude-induced memory impairment [13]. In this study, we determined whether YCl_3_ induced neuronal cell death by regulating apoptotic pathways.

## 2. Materials and Methods

### 2.1. General

Yttrium chloride (purity ≥ 99.99%) was obtained from Sigma-Aldrich (St. Louis, USA). The in situ cell death detection kit, POD (number 1684817), was bought from Roche Applied Science (China). SYBR® Premix Ex Taq™ II (Tli RNaseH Plus) was from TaKaRa (Code: DRR420). DAPK, XIAP, Bcl-2, Bax, caspase-3, Cyto* c*, and GAPDH monoclonal antibodies were purchased from Cell Signaling Technology, Inc. (China).

The study was approved by the Experimental Animal Ethics Committee of Gannan Medical College (number 2009006). Adult Wistar rats (120–140 g) were randomly divided into three groups: negative control group (drinking water) and yttrium chloride (12 mmol/L and 24 mmol/L) groups. The dosages of YCl_3_ were recommended by our previous studies (data not shown). The rats were sacrificed for assays after six-month free access to water containing YCl_3_.

### 2.2. Immunohistochemistry

Tissue microarray (TMA) consisting of brain cerebral cortex tissue was constructed. Formalin-fixed and paraffin-embedded cerebral cortex section was dewaxed in xylene and graded alcohols, hydrated, and washed in PBS. After pretreatment in a microwave oven, endogenous peroxidase was blocked by 3% hydrogen peroxide in methanol for 20 min, followed by avidin-biotin inhibition using a biotin-blocking kit (DAKO, Germany). Slides were then incubated with DAPK, XIAP, Bcl-2, Bax, caspase-3, Cyto *c*, and GAPDH antibodies, overnight in a moist chamber at 4°C, washed in PBS, and incubated with biotinylated goat anti-rat antibodies (1 : 2000, Proteintech Group). Slides were developed with DAB and counterstained with hematoxylin. Image-Pro Plus 6.0 software (Media Cybernetics, United States) was employed for evaluation and the results were presented as mean optical density (MOD) values.

### 2.3. In Situ Cell Apoptosis Detection

TUNEL staining was performed according to the in situ cell death detection kit protocol. Briefly, Brain cerebral cortex tissues can be pretreated as immunohistochemistry. The sections were incubated with the TUNEL labeling solution for 60 min at 37°C, and stained with POD. The images observation and analysis were referred to the immunohistochemistry.

### 2.4. Gene Expression Analysis

Total RNA was extracted from fresh brain cerebral cortex tissue using the Trizol reagent (Invitrogen, USA) according to the manufacturer's instructions. First-strand cDNA was generated by reverse transcription of 5 *μ*g RNA samples. Real-time PCR was performed using a Real-Time PCR System (Applied Biosystems, USA). The primers were designed using primer premier 5.0 and the sequences were presented in Table 1. The target gene mRNA expression levels were normalized to the GAPDH expression using the 2^−ΔΔCt^ method.

### 2.5. Western-Blot Analysis

Total protein was extracted from fresh brain cerebral cortex tissue. Then the extracted proteins were applied to a 12% SDS-polyacrylamide gel for electrophoresis. The proteins were transported to PVDF membrane and incubated with DAPK, XIAP, Bcl-2, Bax, caspase-3, Cyto* c*, and GAPDH antibodies, followed by incubation with HRP-conjugated anti-mouse IgG for 1 h. Proteins were detected using enhanced chemiluminescence reagent (Pierce, USA) according to the manufacturer's protocol.

### 2.6. Statistical Analyses

All of the results were analyzed using one-way ANOVA to examine mean differences between groups.* P* values < 0.05 were considered as statistically significant.

## 3. Results

### 3.1. YCl_3_ Promoted Neuron Cells Death In Vivo

**Figure 1:**
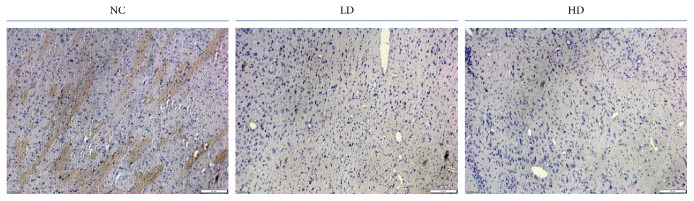
YCl_3_ promoted neuron cells death in vivo. NC: negative control; low dose (LD): 12 mmol/L; high dose (HD): 24 mmol/L. The quantification bar for the MOD of positive stain cells is expressed as means ± SD, *n* = 5. ^*∗*^*P* < 0.05, refers to statistical significance, yttrium chloride group versus control group.

To investigate the toxicity of YCl_3_ in rat brain, the cerebral cortex tissues were prepared for TUNEL staining. As indicated in Figure 1, YCl_3_ treatment promoted neuron cells death in a dose-dependent manner. After treatment with 24 mmol/L YCl_3_, the apoptotic ratio of neuron cells was significantly increased, compared with that in the negative control group.

### 3.2. YCl_3_ Promoted the Expression of Apoptosis Factors In Situ

**Figure 2:**
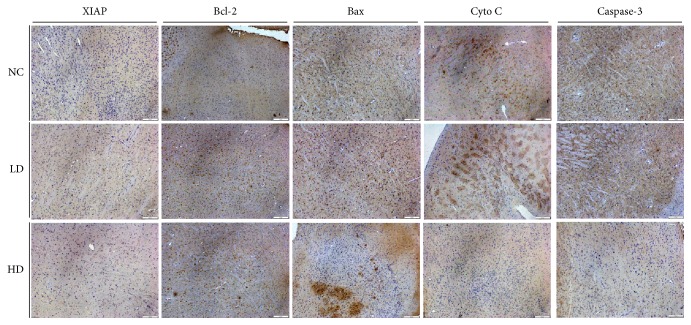
YCl_3_ promoted the expression of apoptosis factors in situ. NC: negative control; low dose (LD): 12 mmol/L; high dose (HD): 24 mmol/L. The quantification bar for the MOD of positive stain cells is expressed as means ± SD, *n* = 5. ^*∗*^*P* < 0.05, refers to statistical significance, yttrium chloride group versus control group.

To prove the effects of YCl_3_ on cell apoptosis in neuron cells, we investigated the expression of apoptosis factors, including XIAP, Bcl-2, Bax, caspase-3, and Cyto* c*, by immunohistochemistry in situ (Figure 2). As a result, YCl_3_ treatment significantly promoted upregulation of apoptosis-related factors Bax, caspase-3, and Cyto *c* and downregulation of antiapoptosis-related factors XIAP and Bcl-2 dose-dependently. Thus, YCl_3_ promoted brain damaged by induction of apoptosis pathways.

### 3.3. YCl_3_ Activated the Genes Expression of Apoptosis Pathways

**Figure 3:**
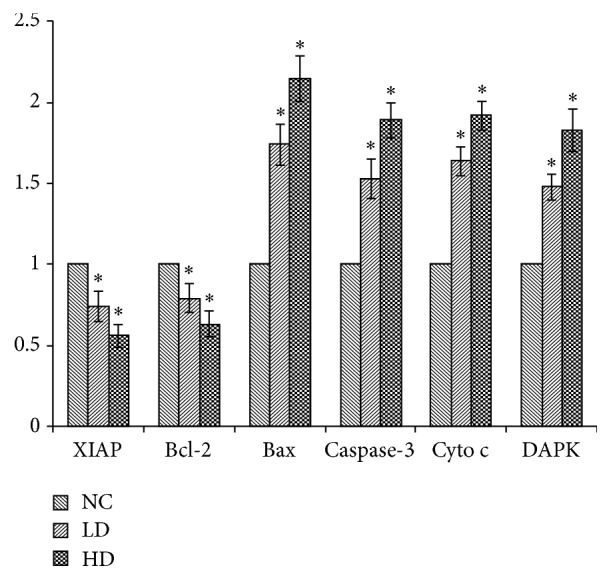
The change in genes expression of apoptosis-related pathways induced by YCl3. Values are expressed as means ± SD, *n* = 6. ^*∗*^*P* < 0.05 as compared with the control group.

To determine whether YCl_3_ exhibited proapoptosis activity by regulating the gene expression of apoptotic pathways, we quantified the mRNA expression of* DAPK*,* XIAP*,* Bcl-2*,* Bax*,* caspase-3*, and* Cyto c* by real-time PCR (Figure 3). YCl_3_ treatment upregulated* DAPK*,* Bax*,* caspase-3*, and* Cyto c* genes expression, while it significantly downregulated the genes expression of* XIAP* and* Bcl-2*.

### 3.4. YCl_3_ Upregulated the Protein Expression of Apoptotic Pathways

**Figure 4:**
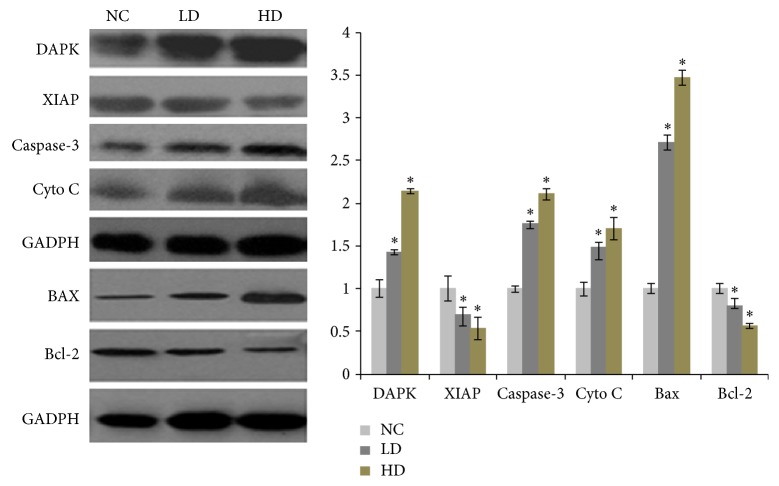
The change in proteins expression of apoptosis-related pathways induced by YCl3. Values are expressed as means ± SD, *n* = 6. ^*∗*^*P* < 0.05 as compared with the control group.

To further determine the changes of apoptotic pathways induced by YCl_3_ treatment in protein level, western-blot assays were applied (Figure 4). With similar trend to their change in gene expression, the apoptotic expressions of DAPK, Bax, caspase-3, and Cyto *c* proteins were found to be upregulated, while YCl_3_ treatment significantly downregulated XIAP and Bcl-2 protein expression, compared with those in the control group.

## 4. Discussion

One of the outstanding characters of neurodegenerative diseases is aberrant neuronal death, which can be induced by stroke, trauma, or other detrimental stimulants, such as REEs [14]. Our previous epidemiological investigation found that the health of the local residents in the south of Jiangxi Province in China, where is the place rich in REEs, has been greatly impacted and damaged. The distribution of yttrium in rats has been determined and showed that it can be severely accumulated in stomach, lungs, kidney, spleen, and thighbone as time goes on (these data are published in Chinese) [15]. To determine whether yttrium induced the neuronal cell death, we further tested the proapoptotic role of YCl_3_ in neuronal cells.

Our results demonstrated that YCl_3_ treatment greatly promoted neuron cells death. The chromosomal DNA fragments are the basic features of neuron cells death, which can be detected by TUNEL analysis [16]. Our study showed that the number of TUNEL-positive neuron cells we quantified was significantly altered in vivo by YCl_3_ treatment. To further prove the neuronal cells apoptosis induced by YCl_3_, immunohistochemistry was employed to verify the activation of apoptosis signaling pathways in situ. The caspase family of proteases is the crucial player responsible for the deliberate disassembly of the cells into apoptotic bodies. Caspases are inactive monomeric proenzymes that require dimerization and are activated by proteolytic cleavage [17, 18]. But caspase-8 and caspase-9 are activated by dimerization and not by proteolytic cleavage [19]. Once activated, caspases can cleave and activate other caspases, resulting in a positively accelerated feedback loop of activation [20–22]. Caspase-3 and caspase-9 are in the pivotal junctions in apoptosis signaling pathways. Caspase-9 activates disassembly in response to agents that stimulates the release of Cyto *c* from mitochondria. Caspase-3 appears to amplify the initiation signals of caspase-9 into full-fledged commitment to disassembly [23]. YCl_3_ treatment effectively initiated the activation loop and significantly upregulated the activation of caspase-3 and Cyto* c*, leading to initiation of neuronal cell apoptosis.

Bcl-2 family proteins are the crucial players in caspases activation and apoptosis regulation. Bcl-2, an antiapoptosis protein, has been demonstrated to prevent or delay the initiation of apoptosis induced by a variety of stimuli through prevention of the release of mitochondria activators of caspases [24]. Specifically, Bcl-2 may associate with the apoptosis activating factor-1 (Apaf-1) to prevent the release of Cyto *c* and the activation of caspase-3 and caspase-9 [25]. Another important feature of Bcl-2 family is to form a homo- or heterodimers, neutralizing the competition between these proteins. Bcl-2 can form a heterodimer with Bax, a proapoptotic protein, leading to restraining their proapoptotic activity [26, 27]. The interaction of complexes between antiapoptosis proteins and proapoptosis proteins demonstrates a simple competition model for apoptotic regulation [28]. YCl_3_ promoted neuronal cells apoptosis might be association the imbalance of complex between Bcl-2 and Bax, as indicated by upregulation of Bax and downregulation of Bcl-2. Unfortunately, we did not determine the amount changes of Bcl-2 and Bax, respectively, in their complex.

XIAP, a member of IAP family with baculovirus IAP repeat (BIR) domain, has been shown to bind to and inhibit caspase-3 and caspase-7 via the BIR2 linker region, leading to alternation of cellular localization and blockage of the caspases docking to their substrates [29]. In addition, XIAP has been demonstrated to associate with the active caspase-9/Apaf-1 complex via BIR3 domain, leading to opposing effects on the caspase activity and apoptosis [30]. However, caspase-3 can cleave XIAP in a negative feedback loop to sensitize cells to apoptosis [31]. In this study, YCl_3_ treatment downregulated XIAP at mRNA and protein levels, leading to upregulation of caspase-3 and apoptosis. DAPK, a calcium/calmodulin-regulated protein kinase, has been implicated in modulating cell apoptosis induced by stimuli. DAPK can promote caspase-linked apoptosis and p53-regulated cell death. It has been showed that DAPK is involved in neuronal cell death, and an inhibitor of DAPK can significantly ameliorate brain injury after ischemic stroke [32]. Significantly, the expression of DAPK in cerebral cortex tissues was upregulated by YCl_3_ treatment. However, the potential mechanism is still unclear.

Conclusively, YCl_3_ promoted neuronal cell apoptosis by upregulating the proapoptotic factors Bax, caspase-3, Cyto* c*, and DAPK and downregulating the antiapoptotic factors Bcl-2 and XIAP.

## Figures and Tables

**Table 1:** Primer sequences used in the study for real-time PCR.

Gene	Primer sequence (5′ → 3′)	
	Sense	Antisense
DAPK	TGAGAATGTGAGCGTGAGGAG	GAGCAGTGTAGGTGGTGAGAC
XIAP	TCTACTACACGGGGATTGATGA	CGAAGAAGCAGTTGGGAAAG
Bcl-2	GGTGGACAACATCGCTCTG	ACAGCCAGGAGAAATCAAACA
Bax	GCGATGAACTGGACAACAAC	GCAAAGTAGAAAAGGGCAACC
Cyto *c*	AGGCTGCTGGATTCTCTTACA	GTCTGCCCTTTCTCCCTTCT
Casp3	ACTGGACTGTGGCATTGAGA	AATTTCGCCAGGAATAGTAACC
GAPDH	TGGGTAGAATCATACTGGAACATGTAG	AGGGCTGCCTTCTCTTGTGAC
